# Supplementary material for: Identification of hsa_circ_0018905 as a New Potential Biomarker for Multiple Sclerosis
Source: Cells. 2024 Oct 9;13(19):1668. doi: 10.3390/cells13191668 (PMC11475351; doi:10.3390/cells13191668)
Supplement: Supplementary file 1 [file cells-13-01668-s001.zip › Lodde et al. Supplemental Figure.pdf]

Supplemental Figure

Identification of the *hsa_circ_0018905* as new potential biomarker for Multiple Sclerosis

Lodde V[1]., Zarbo I.R[2,3]., Farina G[3]., Masia A[2,4]., Solla P[2,3]., Campesi I[1]., Delogu G[1]., Muroni M.R[2]., Tsitsipatis D[5]., Gorospe M[5]., Floris M[1]., Cucca F[1]., Idda M.L[1].

[1] Department of Biomedical Sciences, University of Sassari, Sassari, Italy
[2] Department of Medicine, Surgery and Pharmacy, University of Sassari, Italy
[3] Unit of Clinical Neurology, AOU Sassari, Italy
[4] Department of Medical Sciences and Public Health, University of Cagliari, Italy
[5] Laboratory of Genetics and Genomics, National Institute on Aging Intramural Research Program, National Institutes of Health, Baltimore, MD, United States

Corresponding author Dr. Idda Maria Laura: mlidda1@uniss.it

Supplemental Figure S1: Overview of the workflow and different cohorts used in the study.

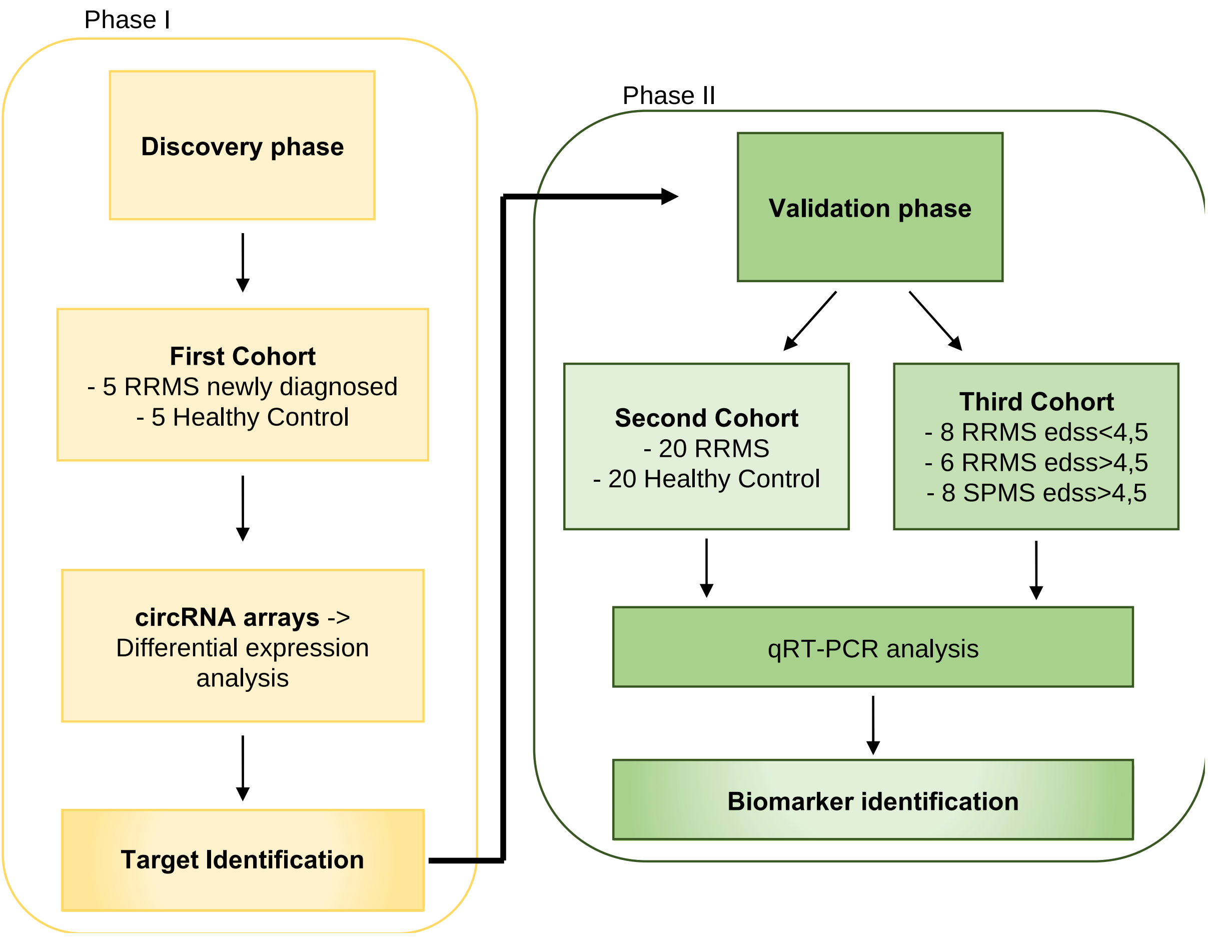

Supplemental Figure S2: *CircRNAs chromosome distribution.*
The identified differentially expressed circRNAs were distributed on all chromosomes. Upregulated circRNAs are represented with red line and down regulated circRNAs are in green.

A

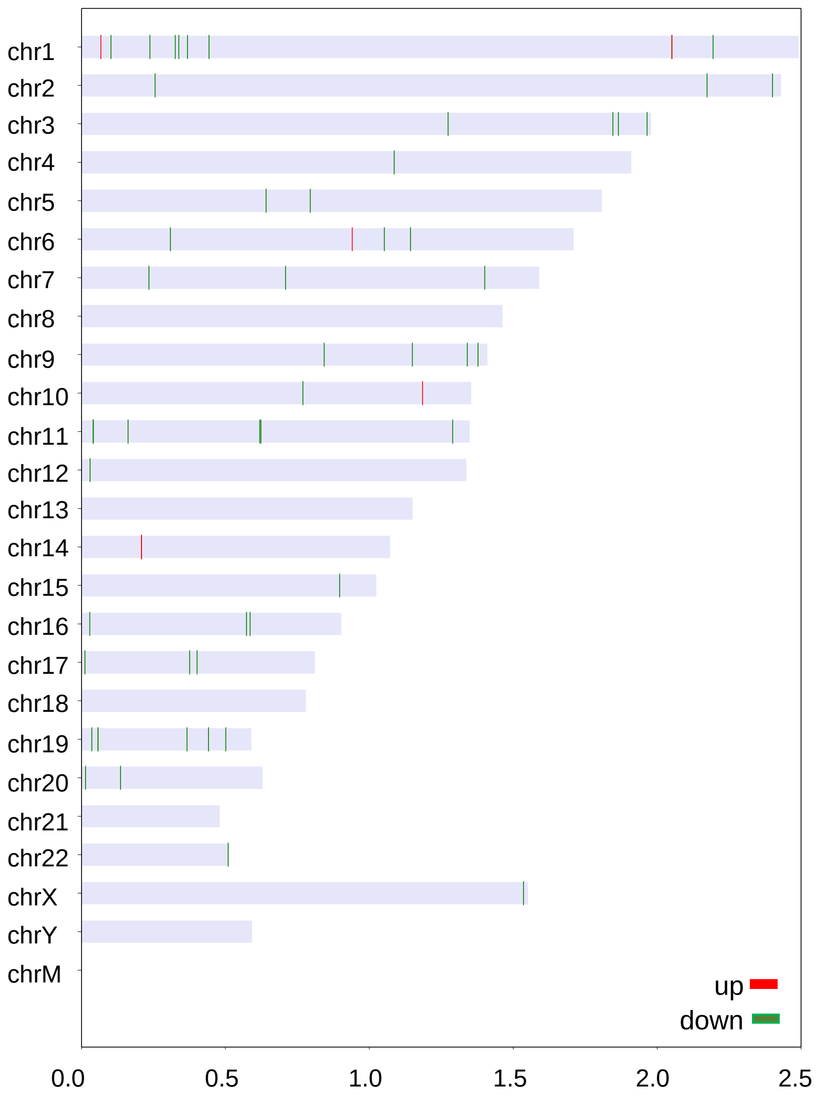

Supplemental Figure S3: *Validation of the circRNAs parental linear transcript in PBMCs of MS patients with different disease severity.*

A)Expression levels in PBMCs of the circRNAs *parental linear transcript* in MS with different disease severity were measured by qPCR analysis. Gene expression levels were normalized to *GAPDH mRNA* levels. Data represent the means and standard deviation (+SD) from at least three independent experiments. *P < 0.05, **P < 0.01, ***P < 0.001.

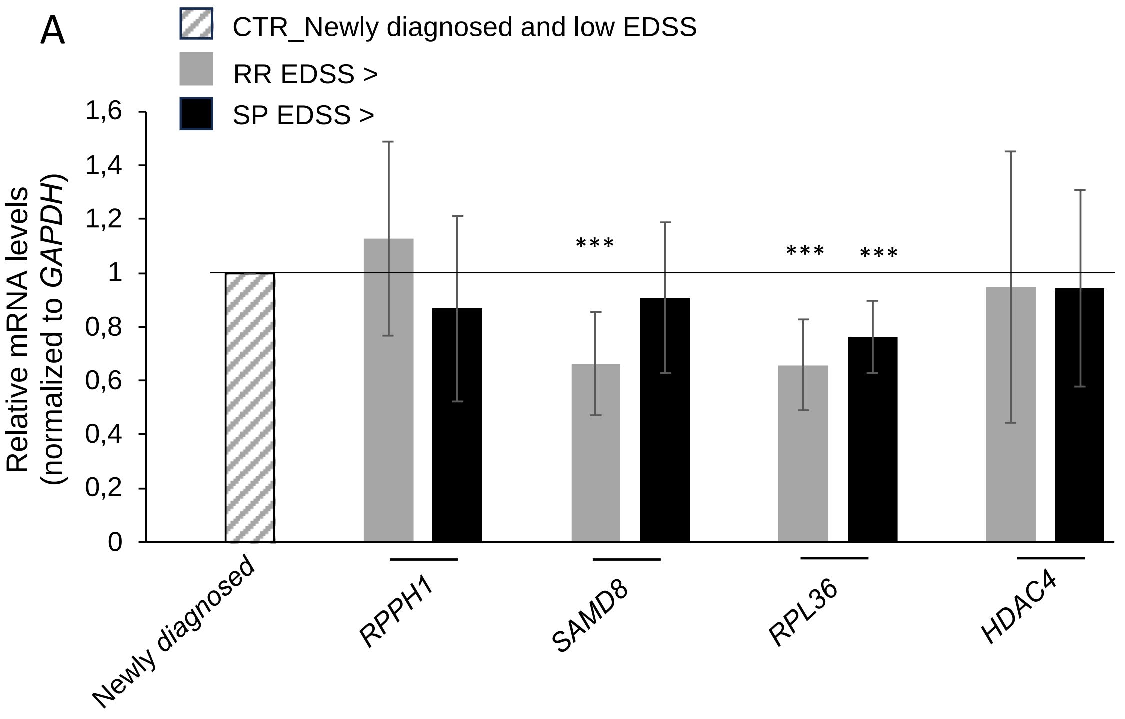

Supplemental Table S1: Complete list of dysregulated circRNAs

(attached in the excel file)
